# Reduced-order modeling of nonlinear multiscale industrial systems via sparse regression in latent representations

**DOI:** 10.1038/s41598-026-53408-4

**Published:** 2026-05-20

**Authors:** Dongni Jia, Xiaofeng Zhou, Shuai Li, Haibo Shi, Linzhi Li

**Affiliations:** 1 Shenyang Institute of Automation, Chinese Academy of Sciences, Shenyang, China; 2 University of Chinese Academy of Sciences, Beijing, China

**Keywords:** Reduced-order modeling, Multiscale nonlinear systems, Sparse regression, Latent representations, Block Hankel embedding, Engineering, Mathematics and computing

## Abstract

Data-driven modeling of nonlinear industrial processes is often complicated by heterogeneous temporal dynamics, measurement noise, and fixed-rate data acquisition. Under such conditions, direct regression on raw time-series data may become sensitive to sampling imbalance and fast transient behavior, leading to degraded predictive performance. This work proposes a structured reduced-order modeling framework for constructing compact and numerically stable predictive surrogates. The approach integrates adaptive resampling to redistribute temporal information, spline-based smoothing for stable derivative estimation, delay embedding to incorporate short-term temporal structure, kernel-based dimensionality reduction to extract dominant patterns, and sparse regression in a latent coordinate space to obtain parsimonious dynamical models. Within this framework, sparsity is used primarily to control model complexity in the reduced representation rather than to recover explicit governing equations. The method is evaluated on two benchmark reactor systems and a real grinding–classification process using chronological train/test splits and multi-step rollout prediction. The results indicate that the proposed approach can improve predictive robustness and numerical stability compared with direct sparse regression and its partial variants, particularly in the presence of multiscale temporal behavior and moderate measurement noise. The framework provides a practical strategy for predictive reduced-order modeling under realistic industrial data constraints. Its design emphasizes stability and compactness of the learned dynamics, while acknowledging that the resulting models are defined in a latent representation and are not intended as exact reconstructions of physical governing equations.

## Introduction

Nonlinear industrial processes often exhibit heterogeneous temporal responses arising from coupled physical, chemical, and operational mechanisms. In many practical systems, different state variables evolve on distinct characteristic time scales. For instance, thermal variables in reactor systems may respond rapidly, whereas concentration dynamics evolve more gradually; similarly, short-term equipment fluctuations in mineral processing circuits may interact with slower production-level variations. In this work, the term *multiscale* is used in an operational sense to describe such heterogeneous temporal behavior under fixed-rate measurement conditions, rather than asymptotically separated scales in the classical sense. This temporal heterogeneity, together with measurement noise and sensor-imposed sampling constraints, poses fundamental challenges for data-driven modeling and prediction.

Data-driven modeling of industrial dynamics serves different objectives depending on the application context. While some approaches aim to recover explicit governing equations, many practical applications–such as monitoring, simulation, and control–prioritize compact and numerically stable predictive models under realistic data constraints. Accordingly, this work focuses on constructing reduced-order surrogate models that capture dominant temporal behavior without aiming at exact reconstruction of physical laws in the original variables. In such settings, reliable multi-step prediction is often more critical than symbolic interpretability.

Machine learning techniques, including neural networks, have demonstrated strong approximation capability for nonlinear time-series data. However, when the modeling objective emphasizes compactness, numerical conditioning, and controlled model complexity, unrestricted black-box models may suffer from overparameterization and sensitivity to sampling imbalance. First-principles modeling and classical identification methods remain effective when sufficient mechanistic knowledge is available^1–4^, but are often impractical for complex industrial systems with limited prior information.

Sparse regression methods, such as Sparse Identification of Nonlinear Dynamics (SINDy)^5,6^, provide a structured approach for constructing compact dynamical representations by promoting sparsity in candidate function libraries^7–10^. Although commonly associated with equation discovery, sparsity can also serve as a regularization mechanism that improves numerical robustness^11^. However, when applied directly to industrial multivariate time-series data, sparse regression in the original state space is often sensitive to noisy differentiation and sampling imbalance between fast and slow dynamics, which may lead to unstable model identification.

To address heterogeneous temporal behavior, feature-based approaches have also been explored. For example, Slow Feature Analysis (SFA)^12,13^ extracts slowly varying components by minimizing temporal variation, and has been widely used in time-series representation learning^14,15^. Nevertheless, such methods are primarily descriptive and do not directly yield predictive dynamical models governing system evolution.

Reduced-order modeling provides a complementary perspective by emphasizing low-dimensional representations that capture dominant system behavior while reducing computational complexity^16–20^. Operator-based viewpoints further suggest that nonlinear dynamics may admit approximately linear evolution in appropriately constructed feature spaces^21,22^. Despite these advances, constructing stable reduced-order models from industrial data remains challenging due to the combined effects of temporal heterogeneity, sampling imbalance, and measurement noise^23,24^. In particular, slowly varying regimes are often densely sampled, whereas transient dynamics are under-represented, leading to ill-conditioned regression problems.

To address these challenges, this work develops a structured data-driven reduced-order modeling framework tailored to industrial time-series data with heterogeneous temporal responses. The proposed pipeline integrates adaptive resampling to mitigate sampling imbalance, spline-based smoothing to stabilize derivative estimation, delay embedding via block Hankel matrices to incorporate temporal correlations, kernel-based dimensionality reduction to extract compact latent representations, and sparse regression in the latent space to construct parsimonious predictive models. Rather than a simple combination of existing techniques, these components are organized to address complementary sources of instability in regression-based modeling.

The approach is evaluated on two chemical reactor systems with distinct temporal characteristics and on a real grinding–classification process with industrial measurements. The results indicate that the proposed framework can improve predictive robustness and numerical stability compared with direct sparse regression in the original state space, particularly in the presence of multiscale temporal behavior and measurement noise.

The main contributions of this study are summarized as follows:

- A reduced-order modeling framework for nonlinear industrial systems with heterogeneous temporal responses, emphasizing compact and numerically stable predictive surrogates;
- A structured preprocessing and representation strategy integrating adaptive resampling, spline smoothing, and delay embedding to improve robustness to noise and sampling imbalance;
- Latent-space sparse regression that enables compact and well-conditioned reduced-order models suitable for efficient prediction;
- Validation on both simulated and real industrial systems, demonstrating improved predictive robustness compared with direct sparse modeling approaches.

For clarity, the principal symbols used throughout the paper are summarized in the Nomenclature section.

## Sparse regression background

Sparse regression provides a structured framework for constructing compact dynamical models from time-series data. Rather than relying on detailed first-principles derivations, it assumes that the evolution of a dynamical system can be approximated by a linear combination of candidate nonlinear functions with relatively few active terms. In this study, sparse regression is introduced as a baseline modeling formulation and as the core regression mechanism employed in the proposed reduced-order pipeline.

Consider a continuous-time dynamical system

1 $$\begin{aligned} \frac{d\boldsymbol{x}}{dt} = F(\boldsymbol{x}), \end{aligned}$$

where $\boldsymbol{x}(t) \in \mathbb {R}^n$ denotes the system state and $F(\cdot )$ is an unknown nonlinear mapping. Given discrete measurements $\{\boldsymbol{x}(t_k)\}_{k=1}^{N}$, the objective is to construct an approximate model of the form

2 $$\begin{aligned} \frac{d\boldsymbol{x}}{dt} \approx \Theta (\boldsymbol{x}) \boldsymbol{\Xi }, \end{aligned}$$

where $\Theta (\boldsymbol{x}) \in \mathbb {R}^{N \times p}$ is a library of candidate functions evaluated on the data, and $\boldsymbol{\Xi } \in \mathbb {R}^{p \times n}$ is a coefficient matrix.

Each column of $\Theta (\boldsymbol{x})$ corresponds to a candidate nonlinear feature, such as polynomial, interaction, or other basis functions. The regression coefficients are determined by solving a regularized least-squares problem,

3 $$\begin{aligned} \min _{\boldsymbol{\Xi }} \left\| \frac{d\boldsymbol{x}}{dt} - \Theta (\boldsymbol{x}) \boldsymbol{\Xi } \right\| _F^2 + \mathscr {R}(\boldsymbol{\Xi }), \end{aligned}$$

where $\Vert \cdot \Vert _F$ denotes the Frobenius norm and $\mathscr {R}(\boldsymbol{\Xi })$ is a sparsity-promoting regularization term. A common choice is the $\ell _1$ penalty,

4 $$\begin{aligned} \mathscr {R}(\boldsymbol{\Xi }) = \lambda \Vert \boldsymbol{\Xi }\Vert _1, \end{aligned}$$

which encourages many coefficients to vanish. More generally, combined penalties such as elastic net regularization may be employed to balance sparsity and numerical stability in the presence of correlated features.

When applied directly in the original state space, sparse regression methods such as SINDy have demonstrated effectiveness in constructing compact dynamical representations, particularly for low-dimensional systems. In this work, sparse regression serves two roles. First, it provides a baseline modeling strategy for comparison. Second, it forms the final regression stage of the proposed framework, where it is applied not in the original coordinates, but in a reduced latent representation constructed through structured preprocessing and dimensionality reduction.

Despite its structural advantages, direct sparse regression on industrial multivariate time-series data faces practical challenges. Estimation of time derivatives from noisy fixed-rate measurements may introduce instability, and regression performed in the original state space can be sensitive to imbalance between rapidly and slowly varying components. These limitations motivate the development of a robustness-oriented reduced-order modeling pipeline described in the next section.

## Model: Framework and implementation

### Overall modeling strategy

Let $\boldsymbol{x}(t) \in \mathbb {R}^{d}$ denote a multivariate industrial time series sampled at discrete time instants $\{t_k\}_{k=1}^{N}$ with fixed sampling interval $\Delta t$. The objective is to construct a compact reduced-order surrogate that reliably captures the dominant temporal evolution of $\boldsymbol{x}(t)$ under practical data constraints, including measurement noise, heterogeneous temporal responses, and fixed-rate sampling.

When sparse regression is applied directly to industrial time-series data, several structural difficulties arise. Uniform sampling tends to over-represent slowly varying regimes while under-resolving transient dynamics, leading to imbalance in the regression problem. Numerical differentiation of noisy measurements further amplifies instability in derivative estimation. In addition, regression in the original state space is often affected by high dimensionality and correlated features, particularly when interactions span multiple characteristic time scales. These factors collectively degrade both numerical conditioning and predictive stability.

**Fig. 1:**
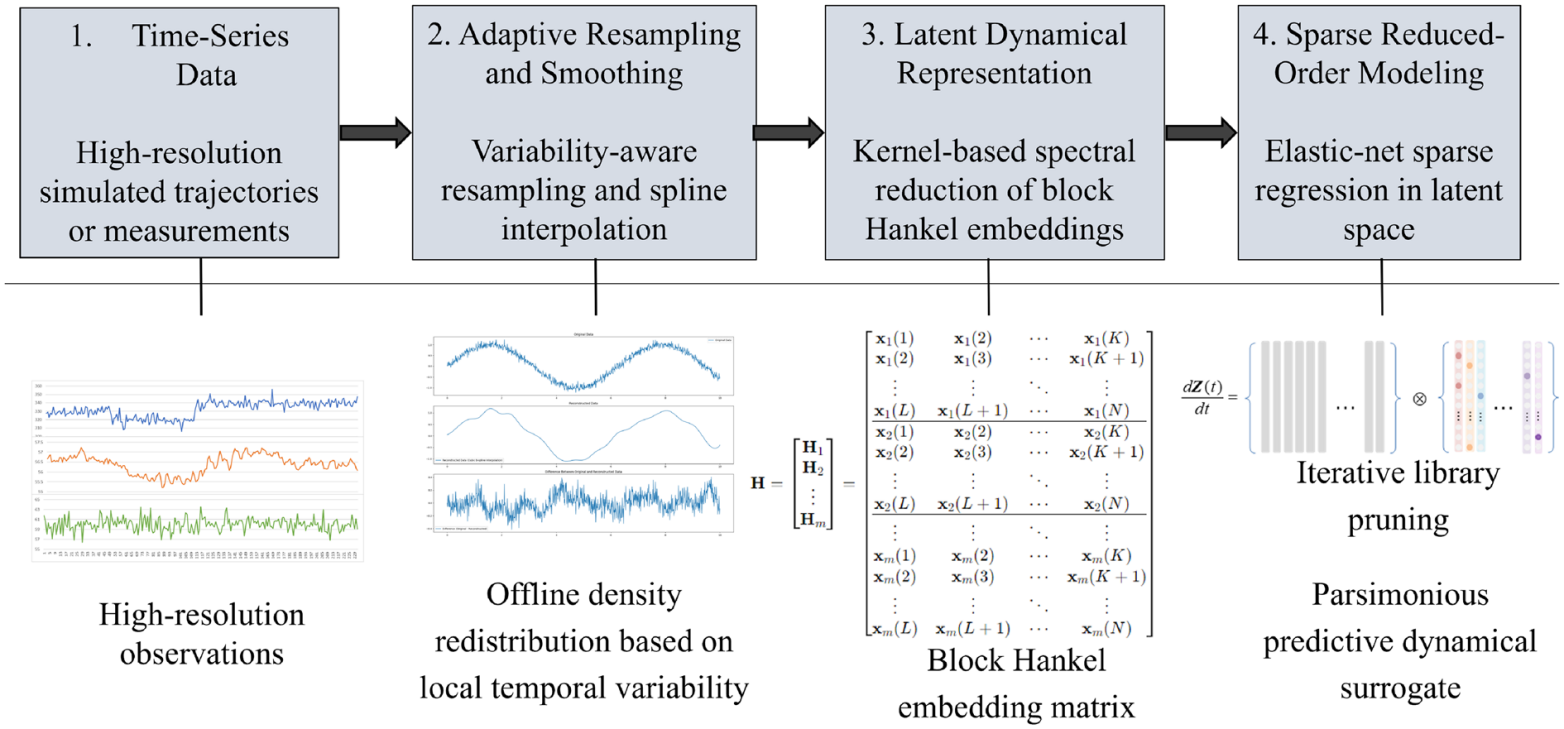
Schematic architecture of the proposed data-driven framework for reduced-order modeling of nonlinear multiscale systems.

The proposed framework addresses these issues through a coordinated sequence of operations. Adaptive resampling redistributes effective data density across temporal regimes, spline-based smoothing improves derivative stability, delay embedding introduces structured temporal information, and kernel-based reduction extracts a compact latent representation with improved conditioning. Sparse regression is then performed in this reduced space to obtain a parsimonious dynamical surrogate. Together, these steps transform the original identification problem into a more stable regression task in a structured representation, as illustrated in Fig. 1.

### Adaptive resampling

Industrial measurements are commonly recorded at a fixed sampling interval $\Delta t$ dictated by sensing and control infrastructure. Under heterogeneous temporal responses, uniform sampling can lead to an imbalanced representation of the dynamics: slowly varying regimes are typically over-represented, whereas transient dynamics remain under-sampled. As a result, the regression objective becomes biased toward fitting low-variation regions, which can degrade accuracy in fast transient phases. To alleviate this imbalance, we employ an *offline adaptive resampling* procedure that redistributes the effective sampling density according to local temporal variability. This step operates directly on the available measurements without introducing new observations or altering the recorded values. Instead, it selects a subset of time instants that better capture rapid changes, resulting in a more balanced representation of temporal variability and improved conditioning of the regression problem.

Let $\{\boldsymbol{x}(t_k)\}_{k=1}^{N}$ denote the observed multivariate time series with $\boldsymbol{x}(t_k)=[x^{(1)}(t_k),\ldots ,x^{(d)}(t_k)]^{\top }\in \mathbb {R}^{d}$. Local variability is assessed using three complementary indicators computed from each scalar component $x^{(j)}(t_k)$. First, a one-step linear extrapolation captures deviation from a local trend:

5 $$\begin{aligned} \hat{x}^{(j)}(t_k) = x^{(j)}(t_{k-1}) + \big (x^{(j)}(t_{k-1})-x^{(j)}(t_{k-2})\big ), \quad k\ge 3, \end{aligned}$$

and the corresponding prediction error is

6 $$\begin{aligned} e_k^{(j)} = x^{(j)}(t_k) - \hat{x}^{(j)}(t_k). \end{aligned}$$

Second, a spectral-variation proxy is computed over a sliding window of length *w* to reflect changes in local oscillatory content. Let $\{x^{(j)}(t_{k-w+1}),\ldots ,x^{(j)}(t_k)\}$ be the windowed segment and $F_k^{(j)}(\cdot )$ its discrete Fourier coefficients; we define

7 $$\begin{aligned} s_k^{(j)} = \sum _{m=1}^{w-1}\left| \,|F_k^{(j)}(m)|^2 - |F_k^{(j)}(m-1)|^2\,\right| . \end{aligned}$$

Third, trend stability is quantified by the variability of local finite-difference slopes. Using

8 $$\begin{aligned} \kappa _i^{(j)}=\frac{x^{(j)}(t_{i+1})-x^{(j)}(t_i)}{\Delta t}, \end{aligned}$$

the trend-instability index over the most recent *l* samples is

9 $$\begin{aligned} \ell _k^{(j)}=\frac{1}{l-1}\sum _{i=k-l+1}^{k-1}\left| \kappa _i^{(j)}-\kappa _{i-1}^{(j)}\right| . \end{aligned}$$

The above indicators are combined into a composite variability score for each component,

10 $$\begin{aligned} I_k^{(j)} = w_1\,|e_k^{(j)}| + w_2\, s_k^{(j)} + w_3\, \ell _k^{(j)}, \end{aligned}$$

and then aggregated into a single multivariate variability measure

11 $$\begin{aligned} I_k = \frac{1}{d}\sum _{j=1}^{d} I_k^{(j)}. \end{aligned}$$

Time instants with large $I_k$ are associated with rapidly varying regimes, whereas persistently small $I_k$ indicates slowly varying intervals. The resampled index set is obtained by preferentially retaining time instants from high-variability segments while sparsifying redundant points in stable regions, yielding a subsequence $\{t_{i_1},\ldots ,t_{i_M}\}$ with $M\le N$.

The weighting coefficients $(w_1,w_2,w_3)$ balance three complementary notions of variability: local trend deviation, short-window spectral fluctuation, and slope inconsistency. In practice, we use normalized indicators so that the three terms are comparable in magnitude, and the weights mainly reflect relative emphasis (e.g., favoring transient detection versus trend consistency). As the procedure is used for density redistribution rather than parameter estimation, moderate perturbations of $(w_1,w_2,w_3)$ primarily affect the aggressiveness of sparsification in slowly varying regions without changing the qualitative selection of transient segments.

**Algorithm 1:**
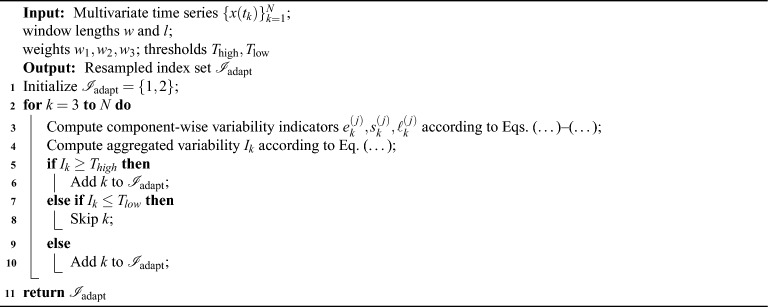
Offline adaptive resampling

### Spline-based smoothing and differentiation

Following adaptive resampling, a continuous representation of the time series is constructed to enable stable numerical differentiation. Let $\{\boldsymbol{x}(t_{i_k})\}_{k=1}^{M}$ denote the resampled multivariate sequence, where $\boldsymbol{x}(t_{i_k}) \in \mathbb {R}^{d}$. Each scalar component $x^{(j)}(t)$ is approximated independently using cubic B-spline interpolation.

Given the discrete observations $\{x^{(j)}(t_{i_k})\}_{k=1}^{M}$, the spline approximation is written as

12 $$\begin{aligned} S^{(j)}(t) = \sum _{m=0}^{q} c_m^{(j)}\, N_{m,3}(t), \end{aligned}$$

where $N_{m,3}(t)$ are cubic B-spline basis functions defined over a prescribed knot sequence and $c_m^{(j)}$ are coefficients determined by interpolation constraints. The resulting function $S^{(j)}(t)$ provides a smooth approximation of the discrete measurements.

Cubic B-splines possess $C^2$ continuity, ensuring that first and second derivatives are continuous across knot intervals. This property enables analytical evaluation of temporal derivatives,

13 $$\begin{aligned} \frac{d x^{(j)}}{dt}(t) \approx \frac{d S^{(j)}}{dt}(t), \end{aligned}$$

which yields numerically stable estimates of $\frac{d\boldsymbol{x}}{dt}$ required for subsequent regression.

The spline stage is introduced exclusively to regularize derivative computation under noisy measurements and uneven sampling density. It does not alter the resampled time instants nor introduce artificial dynamics; rather, it provides a smooth interpolation that preserves the observed trajectory while improving numerical conditioning in the identification stage.

### Block Hankel delay embedding

While spline smoothing stabilizes derivative estimation at individual time instants, regression in the original state space still relies on instantaneous observations $\boldsymbol{x}(t_k) \in \mathbb {R}^{d}$. For multiscale systems, such pointwise representations may inadequately reflect temporal dependencies that extend across multiple sampling steps. To incorporate short-term temporal structure explicitly, we construct a delay-embedded representation using block Hankel matrices.

Let $\{\boldsymbol{x}(t_{i_k})\}_{k=1}^{M}$ denote the resampled sequence. For a prescribed window length *L*, the delay embedding of the *j*-th component $x^{(j)}(t)$ is defined as the Hankel matrix

14 $$\begin{aligned} \mathscr {H}_j = \begin{bmatrix} x^{(j)}(t_{i_1}) & x^{(j)}(t_{i_2}) & \cdots & x^{(j)}(t_{i_P}) \\ x^{(j)}(t_{i_2}) & x^{(j)}(t_{i_3}) & \cdots & x^{(j)}(t_{i_{P+1}}) \\ \vdots & \vdots & \ddots & \vdots \\ x^{(j)}(t_{i_L}) & x^{(j)}(t_{i_{L+1}}) & \cdots & x^{(j)}(t_{i_M}) \end{bmatrix}, \end{aligned}$$

where $P = M - L + 1$. Each column of $\mathscr {H}_j$ collects *L* consecutive observations of the same variable, thereby encoding local temporal evolution over a finite horizon.

To account for cross-variable interactions, the individual Hankel matrices are vertically concatenated to form the block Hankel matrix

15 $$\begin{aligned} \hat{\mathscr {H}} = \begin{bmatrix} \mathscr {H}_1 \\ \mathscr {H}_2 \\ \vdots \\ \mathscr {H}_d \end{bmatrix} \in \mathbb {R}^{dL \times P}. \end{aligned}$$

This construction expands the state dimension from *d* to *dL*, incorporating both intra-variable temporal correlations and inter-variable coupling into a unified structured representation. Through block Hankel embedding, short-term temporal history is explicitly encoded, effectively lifting the dynamics into a higher-dimensional space. In this representation, temporal dependencies are more directly captured, which reduces reliance on instantaneous observations and improves the stability of subsequent regression.

Compared with independent delay embedding or direct regression on stacked time-shifted vectors, the block Hankel formulation yields a compact matrix structure amenable to spectral decomposition. The window length *L* determines the temporal horizon: larger values capture longer memory effects but increase dimensionality, whereas smaller values emphasize short-term dynamics. In practice, *L* is chosen such that $L\Delta t$ aligns with the dominant transient duration, balancing expressiveness and computational cost.

The resulting matrix $\hat{\mathscr {H}}$ is then passed to the kernel-based dimensionality reduction stage, where its structured redundancy is leveraged to obtain a low-dimensional latent representation.

### Kernel-based dimensionality reduction

The block Hankel matrix $\hat{\mathscr {H}} \in \mathbb {R}^{dL \times P}$ constructed in the previous subsection encodes short-horizon temporal information across all variables. However, its dimension *dL* may remain high, particularly when the embedding window *L* is chosen to capture transient dynamics. To obtain a compact representation suitable for regression, we perform a kernel-based spectral reduction on the column space of $\hat{\mathscr {H}}$.

Let $\hat{\mathscr {H}}_k \in \mathbb {R}^{dL}$ denote the *k*-th column of $\hat{\mathscr {H}}$, corresponding to the delay vector centered at time $t_{i_k}$. A Gaussian kernel is used to define pairwise similarities,

16 $$\begin{aligned} K_{kl} = \exp \!\left( -\frac{\Vert \hat{\mathscr {H}}_k - \hat{\mathscr {H}}_l\Vert _2^2}{2\sigma ^2}\right) , \end{aligned}$$

yielding the Gram matrix $\boldsymbol{K} \in \mathbb {R}^{P \times P}$. The Gaussian kernel introduces a nonlinear similarity measure that captures local similarity structure in the delay-embedded space while remaining robust to moderate noise.

To ensure consistency with centered feature representations, the Gram matrix is centered as

17 $$\begin{aligned} \tilde{\boldsymbol{K}} = \boldsymbol{K} - \boldsymbol{1}\boldsymbol{K} - \boldsymbol{K}\boldsymbol{1} + \boldsymbol{1}\boldsymbol{K}\boldsymbol{1}, \end{aligned}$$

where $\boldsymbol{1} = \frac{1}{P}\textbf{e}\textbf{e}^{\top }$ and $\textbf{e} \in \mathbb {R}^{P}$ is the vector of ones.

Spectral decomposition of the centered matrix,

18 $$\begin{aligned} \tilde{\boldsymbol{K}} = \boldsymbol{U}\boldsymbol{\Sigma }\boldsymbol{U}^{\top }, \end{aligned}$$

provides an ordered set of singular values $\{\sigma _i\}_{i=1}^{P}$. The latent dimension *r* is selected such that

19 $$\begin{aligned} \frac{\sum _{i=1}^{r} \sigma _i}{\sum _{i=1}^{P} \sigma _i} \ge \eta , \end{aligned}$$

where $\eta$ is a prescribed energy threshold (set to 0.95 in our experiments). The reduced latent representation is then defined as

20 $$\begin{aligned} \boldsymbol{Z} = \boldsymbol{\Sigma }_r^{1/2} \boldsymbol{U}_r^{\top } \in \mathbb {R}^{r \times P}, \end{aligned}$$

where $\boldsymbol{U}_r$ and $\boldsymbol{\Sigma }_r$ denote the leading *r* eigenvectors and eigenvalues, respectively.

The kernel bandwidth $\sigma$ controls the locality of the similarity measure. Excessively small values may lead to noise-sensitive embeddings, whereas overly large values approach a nearly linear mapping. In practice, $\sigma$ is initialized using the median pairwise distance between columns of $\hat{\mathscr {H}}$ and refined through validation to balance stability and discriminative power.

This kernel-based reduction maps the high-dimensional delay representation into a compact latent coordinate system $\boldsymbol{Z}$ that preserves dominant temporal patterns while suppressing noise and redundant variability. By concentrating the dynamics in a lower-dimensional subspace, it improves numerical conditioning and reduces sensitivity to multicollinearity in the subsequent regression stage.

### Sparse regression in the latent space

Let $\boldsymbol{Z} = [\boldsymbol{z}(t_{i_1}),\ldots ,\boldsymbol{z}(t_{i_P})] \in \mathbb {R}^{r \times P}$ denote the latent coordinate matrix obtained from the previous stage, where $\boldsymbol{z}(t_{i_k}) \in \mathbb {R}^{r}$ represents the reduced state at time $t_{i_k}$. The objective is to identify a compact dynamical model governing the temporal evolution of $\boldsymbol{z}(t)$.

A library of candidate nonlinear functions is constructed in the latent space,

21 $$\begin{aligned} \Theta (\boldsymbol{z}) = \big [\theta _1(\boldsymbol{z}), \ldots , \theta _q(\boldsymbol{z})\big ], \end{aligned}$$

where each $\theta _\ell (\boldsymbol{z})$ denotes a predefined basis function (e.g., polynomial or interaction terms) evaluated on $\boldsymbol{z}(t)$. Using the spline-based derivative estimates, the regression problem is formulated as

22 $$\begin{aligned} \min _{\boldsymbol{\Xi }} \left\| \dot{\boldsymbol{Z}} - \Theta (\boldsymbol{Z})\,\boldsymbol{\Xi } \right\| _F^2 + \lambda _1 \Vert \boldsymbol{\Xi }\Vert _1 + \lambda _2 \Vert \boldsymbol{\Xi }\Vert _F^2, \end{aligned}$$

where $\dot{\boldsymbol{Z}} \in \mathbb {R}^{r \times P}$ denotes the estimated time derivatives of the latent states computed from the spline-smoothed trajectories. And $\boldsymbol{\Xi } \in \mathbb {R}^{q \times r}$ is the coefficient matrix.

The $\ell _1$ penalty promotes sparsity in the selected terms, encouraging a parsimonious representation of the latent dynamics, while the $\ell _2$ penalty mitigates instability arising from correlated features in the library. This elastic net formulation generalizes the classical $\ell _1$-based sparse identification framework and improves numerical robustness in the presence of noise and multicollinearity. The regularization parameters $(\lambda _1,\lambda _2)$ are selected via cross-validation on the training data to balance model complexity and predictive accuracy.

The resulting system

23 $$\begin{aligned} \dot{\boldsymbol{z}}(t) = \Theta (\boldsymbol{z}(t))\,\boldsymbol{\Xi }, \end{aligned}$$

defines a reduced-order dynamical surrogate in the latent coordinate space. This model is integrated forward in time for prediction and simulation. The identified dynamics are defined in the learned latent representation and are intended to provide stable predictive surrogates rather than explicit recovery of governing equations in the original physical variables.

### Hyperparameter summary

Table 1 summarizes the key hyperparameters introduced in the modeling pipeline together with their roles and selection principles.

**Table 1:** Summary of key hyperparameters in the proposed modeling framework.

**Symbol**	**Module**	**Role and selection principle**
*w*	Adaptive resampling	Length of sliding window for spectral variation computation; chosen to cover local transient duration.
*l*	Adaptive resampling	Length of slope window for trend stability assessment; selected relative to sampling interval $\Delta t$.
$w_1, w_2, w_3$	Adaptive resampling	Weights in composite variability score $I_k$; balance trend deviation, spectral fluctuation, and slope instability.
*L*	Delay embedding	Embedding window length; determines temporal horizon $L\Delta t$ captured in block Hankel matrix.
$\sigma$	Kernel reduction	Gaussian kernel bandwidth; initialized via median pairwise distance heuristic and refined by validation.
*r*	Kernel reduction	Retained latent dimension; selected based on cumulative spectral energy threshold (e.g., 95%).
$\lambda _1, \lambda _2$	Sparse regression	Elastic-net regularization parameters; selected via cross-validation to balance sparsity and numerical stability.

## Applications and discussion

To assess the proposed pipeline as a *predictive reduced-order modeling* tool under realistic industrial data constraints, we evaluate it on two simulated chemical reactor systems and one real-world grinding–classification process. All experiments are designed around multivariate time-series inputs sampled at fixed intervals, where heterogeneous temporal responses and measurement noise may coexist. Unless otherwise stated, each dataset is split into disjoint training and testing subsets, and all reported results are computed on the test set using multi-step rollout prediction over the same forecast horizon.

To examine robustness, we consider additive measurement noise at prescribed levels. Specifically, for each measured channel $x^{(j)}(t_k)$ we form a corrupted observation

24 $$\begin{aligned} \tilde{x}^{(j)}(t_k)=x^{(j)}(t_k)+\epsilon \,\sigma _j\,\eta _k,\qquad \eta _k\sim \mathscr {N}(0,1), \end{aligned}$$

where $\sigma _j$ is the standard deviation of the clean signal of the *j*-th channel over the training portion and $\epsilon \in \{0,0.01,0.10\}$ denotes the noise ratio. To ensure transparent comparison, we define two restricted pipeline variants used as baselines. AdS-SINDy applies only the adaptive resampling stage prior to standard sparse regression, whereas Hankel-SINDy performs regression on delay-embedded coordinates without kernel-based reduction or latent-space modeling. The proposed method is compared against SINDy and these two variants, using consistent training/testing protocols. For all case studies, hyperparameters are selected using a consistent validation protocol within the training data. Model selection is performed exclusively on the training subset, and the testing data are not used during parameter tuning. Baseline methods are tuned under the same validation procedure to ensure fair comparison across all experiments. Unless otherwise specified, the selection principles summarized in Table 1 are applied uniformly to all systems. In addition to accuracy, we emphasize qualitative behavior in transient segments and long-horizon stability, as these properties are critical for reduced-order surrogates intended for efficient prediction and simulation in practice.

### Isothermal batch reactor with multiple reactions

We first consider an isothermal, constant-volume batch reactor governed by the reaction network

25 $$\begin{aligned} \textrm{A} \rightleftarrows \textrm{B} \rightarrow \textrm{C}, \end{aligned}$$

in which the reversible reaction between species A and B evolves on a faster time scale than the irreversible conversion of B to C. The corresponding mass-balance equations are

26 $$\begin{aligned}&\left\{ \begin{array}{l} \frac{dC_{A}}{dt} = -k_{1}C_{A} + k_{-1}C_{B}, \\ \frac{dC_{B}}{dt} = k_{1}C_{A} - k_{-1}C_{B} - k_{2}C_{B}, \\ \frac{dC_{C}}{dt} = k_{2}C_{B}, \end{array} \right. \end{aligned}$$

27 $$\begin{aligned}&C_A(0)+C_B(0) =\text {constant},\quad C_C(0)=0 \end{aligned}$$

where $C_i$ denotes the concentration of species *i*. The kinetic parameters are listed in Table 2. With $k_1=5.0~\textrm{h}^{-1}$ and $k_2=0.5~\textrm{h}^{-1}$, the characteristic time scales associated with the fast reversible reaction ($\tau _{\textrm{fast}}\approx 1/k_1=0.2~\textrm{h}$) and the slower conversion to species C ($\tau _{\textrm{slow}}\approx 1/k_2=2~\textrm{h}$) differ by approximately one order of magnitude, leading to distinct transient and long-term behaviors.

**Data generation and evaluation protocol.** Reference trajectories are generated by numerically integrating Eq. (26) with a sufficiently small internal integration step ($10^{-6}$ h) to obtain high-fidelity ground truth. The observation signals used for model identification are sampled at a fixed interval of 0.1 h over a horizon of 0–10 h, consistent with practical sensor constraints. Simulation and observation settings are summarized in Tables 3. Two representative initial conditions are considered. For each initial condition, the first $60\%$ of the trajectory is used for training and the remaining $40\%$ for multi-step rollout evaluation.

Additive Gaussian noise is introduced according to the protocol described at Eq. (24), with noise ratios $\epsilon \in \{0,0.01,0.10\}$ applied independently to each measured concentration channel. All reported prediction errors correspond to the test segment only.

**Table 2:** Parameter Values for Example 1.

Parameter	$k_1$	$k_{-1}$	$k_2$	Constant
Value	$5.0 \, \text {h}^{-1}$	$1.0 \, \text {h}^{-1}$	$0.5 \, \text {h}^{-1}$	$9.0 \, \text {mol/m}^{3}$

**Table 3:** Simulation and observation settings for example 1.

Variable	Sampling interval	Step size
$C_{{A}}(0)$	$0 \, \text {mol m}^{-3}$ to $9.0 \, \text {mol m}^{-3}$	$1.0 \, \text {mol m}^{-3}$
*t* (Time)	$0.0 \, \text {hr}$ to $10.0 \, \text {hr}$	$10^{-6} \, \text {hr}$
Sampling Output Time	$0.0 \, \text {hr}$ to $10.0 \, \text {hr}$	$0.1 \, \text {hr}$

**Predictive performance.** For this system, representative hyperparameter values are $L=15$, $r=4$, with the kernel bandwidth $\sigma$ initialized via the median-distance heuristic and refined by validation. The elastic-net parameters $(\lambda _1,\lambda _2)$ are determined by cross-validation within the training data.

**Fig. 2:**
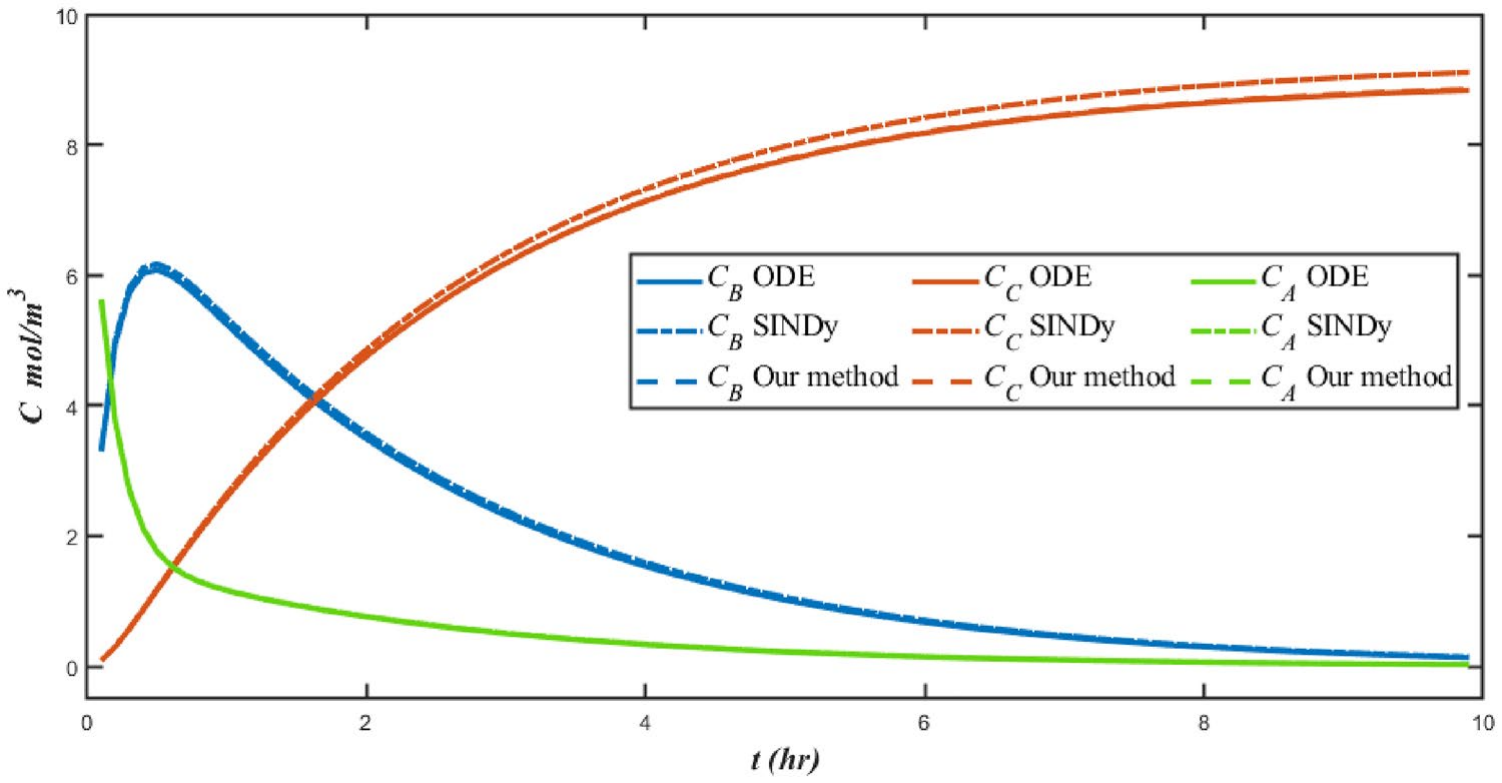
Long-horizon rollout prediction under clean (noise-free) simulated data for the isothermal batch reactor. Ground truth trajectories are compared with predictions obtained using the proposed method and baseline SINDy-based approaches.

Figure 2 shows representative multi-step rollout predictions obtained using the proposed pipeline and baseline approaches. Although this reaction system is linear, it provides a controlled setting to examine the influence of temporal scale separation under fixed-rate sampling. Standard SINDy applied directly to the raw measurements exhibits increasing deviation during the fast transient phase when sampling is relatively coarse. In contrast, the proposed approach maintains stable tracking of both the fast exchange between $C_A$ and $C_B$ and the slower accumulation of $C_C$.

**Fig. 3:**
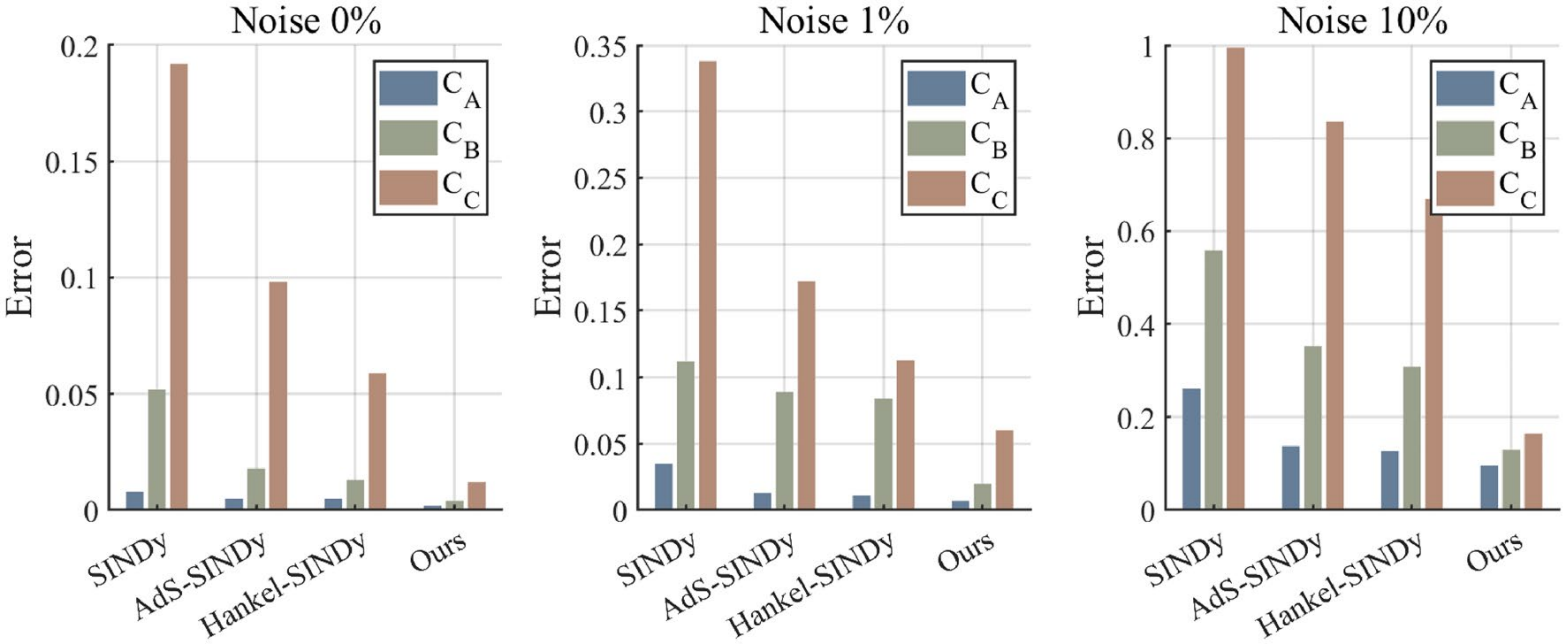
Error comparison of different identification methods under different noise levels for the isothermal batch reactor.

Quantitative results are summarized in Table 4 and Fig. 3. Across both initial conditions and all noise levels, performing sparse regression in the kernel-based delay-embedded latent space yields lower prediction errors than standard SINDy and its partial variants (adaptive-sampling SINDy and Hankel-SINDy). The improvement is particularly pronounced under $10\%$ noise, where direct regression on raw variables becomes increasingly sensitive to measurement perturbations.

**Table 4:** Error comparison for different methods under different initial conditions.

Method	Error (noise 0%)			Error (noise 1%)			Error (noise 10%)		
	$C_A$	$C_B$	$C_C$	$C_A$	$C_B$	$C_C$	$C_A$	$C_B$	$C_C$
Initial Condition 1									
SINDy	0.006	0.048	0.187	0.030	0.105	0.327	0.257	0.549	0.983
AdS-SINDy	0.004	0.014	0.092	0.011	0.082	0.164	0.131	0.346	0.824
Hankel-SINDy	0.004	0.010	0.055	0.009	0.080	0.107	0.122	0.301	0.657
**ours**	**0.001**	**0.002**	**0.009**	**0.005**	**0.017**	**0.055**	**0.091**	**0.122**	**0.156**
Initial Condition 2									
SINDy	0.008	0.052	0.192	0.035	0.112	0.338	0.262	0.558	0.995
AdS-SINDy	0.005	0.018	0.098	0.013	0.089	0.172	0.138	0.353	0.836
Hankel-SINDy	0.005	0.013	0.059	0.011	0.084	0.113	0.127	0.309	0.669
**ours**	**0.002**	**0.004**	**0.012**	**0.007**	**0.020**	**0.060**	**0.096**	**0.130**	**0.165**

While the present example does not involve strong nonlinearities, it highlights the numerical implications of multiscale temporal behavior under fixed sampling. More nonlinear and coupled dynamics are examined in the subsequent CSTR case study.

### Non-isothermal CSTR with jacket

**Fig. 4:**
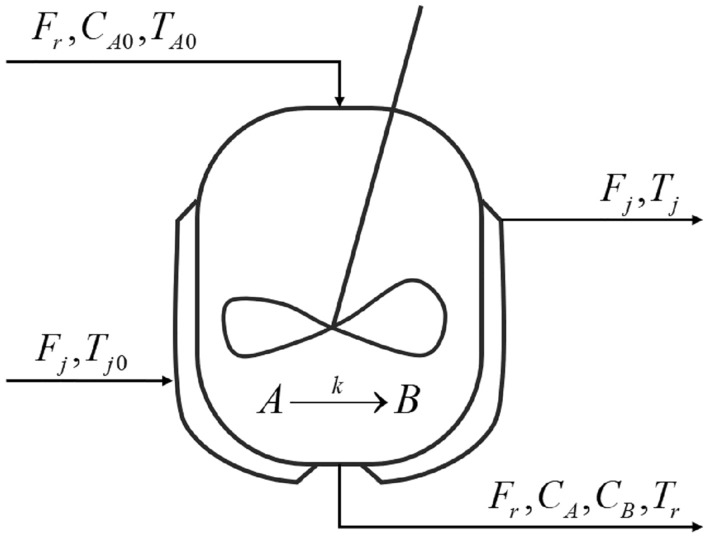
A continuous-stirred tank reactor with jacket.

The second case study considers a non-isothermal continuous stirred tank reactor (CSTR) with jacket cooling, in which an irreversible exothermic reaction $A \rightarrow B$ takes place. A schematic diagram is shown in Fig. 4^25,26^.

The coupled mass and energy balances are given by

28 $$\begin{aligned} \left\{ \begin{aligned} V_r \frac{dC_A}{dt}&= F_r (C_{A0} - C_A) + r V_r,\\ V_r \frac{dT_r}{dt}&= F_r (T_{A0} - T_r) + \frac{\Delta H_r}{\rho _m c_{p,m}} r V_r + \frac{UA_r}{\rho _m c_{p,m}}(T_j - T_r), \\ V_j \frac{dT_j}{dt}&= F_j T_{j0} - F_j T_j - \frac{UA_r}{\rho _j c_{p,j}}(T_j - T_r), \\ r&= -k_0 \exp \!\left( \frac{-E}{RT_r}\right) C_A, \end{aligned} \right. \end{aligned}$$

where $C_A$ is the reactant concentration, $T_r$ the reactor temperature, and $T_j$ the jacket temperature. Parameter values are listed in Table 5.

**Table 5:** Parameter values for example 2.

**Variable**	**Meaning**	**Value**	**Variable**	**Meaning**	**Value**
$C_{A0}$	Inlet concentration of reactant A	$3.75 \, \text {kmol/m}^3$	$\rho _j$	Density of the heat transfer fluid	$800.0 \, \text {kg/m}^3$
$T_{A0}$	Inlet temperature of reactant A	$310.0 \, \text {K}$	$c_{p,j}$	Heat capacity of the heat transfer fluid	$0.2 \, \text {kcal/(kg}\cdot \text {K)}$
$V_r$	Reactor liquid volume	$1.0 \, \text {m}^3$	$\Delta H_r$	Reaction enthalpy change	$5.4 \times 10^4 \, \text {kcal/mol}$
$V_j$	Jacket volume	$0.08 \, \text {m}^3$	*U*	Heat transfer coefficient	$1000.0 \, \text {kcal/(h}\cdot \text {m}^2\cdot \text {K)}$
$F_j$	Jacket circulating fluid flow rate	$20.0 \, \text {m}^3/\text {h}$	$A_r$	Heat exchange area	$6.0 \, \text {m}^2$
$T_{j0}$	Jacket fluid inlet temperature	$357.5 \, \text {K}$	*R*	Gas constant	$1.987 \, \text {kcal/(kmol}\cdot \text {K)}$
$\rho _m$	Density of the reacting liquid	$900.0 \, \text {kg/m}^3$	$k_0$	Pre-exponential factor	$3.36 \times 10^6 \, \text {h}^{-1}$
$c_{p,m}$	Heat capacity of the reacting liquid	$0.231 \, \text {kcal/(kg}\cdot \text {K)}$	*E*	Activation energy of the reaction	$8.0 \times 10^3 \, \text {kcal/kg}$
			$F_r$	Reactor flow rate	$3.0 \, \text {m}^3/\text {h}$

**Multiscale characteristics.** Unlike the linear batch reactor in the previous example, the CSTR exhibits strong nonlinear coupling through the Arrhenius term and heat-transfer interactions. The reaction rate *r* depends exponentially on $T_r$, leading to rapid temperature excursions during ignition-like transients, while concentration and jacket dynamics evolve over comparatively slower time scales governed by flow and heat exchange. Under the parameter settings in Table 5, the dominant thermal transient occurs on a time scale of minutes, whereas concentration adjustment spans a longer interval, resulting in temporally heterogeneous dynamics within the same trajectory.

**Data generation and evaluation protocol.** Reference trajectories are generated using a high-accuracy numerical integrator with internal step size $10^{-6}$ h to obtain ground-truth solutions. Observation signals are sampled at a fixed interval of 0.005 h over a horizon of 0–1 h, consistent with fixed-rate sensing assumptions.

Multiple initial conditions are considered by varying $(C_A(0),T_r(0),T_j(0))$ within the ranges listed in Table 6. For each trajectory, the first $60\%$ of the time horizon is used for model identification, and the remaining $40\%$ for multi-step rollout evaluation. Noise is injected according to the protocol described in Section "Applications and discussion", with noise ratios $\epsilon \in \{0,0.01,0.10\}$ applied independently to each observed channel. All reported errors correspond to predictions on the test segment only.

**Table 6:** Sampling details for the system.

**Variable**	**Meaning**	**Sampling interval**	**Step size**
$C_A(0)$	Concentration of reactant A	$0 \, \text {mol/m}^3$ to $9.0 \, \text {mol/m}^3$	$1.0 \, \text {mol/m}^3$
$T_r(0)$	Reactor temperature	$280 \, \text {K}$ to $370 \, \text {K}$	$10 \, \text {K}$
$T_j(0)$	Jacket temperature	$300 \, \text {K}$ to $390 \, \text {K}$	$10 \, \text {K}$
*t*	Time	$0.0 \, \text {hr}$ to $1.0 \, \text {hr}$	$10^{-6} \, \text {hr}$
Sampling Output Time	-	$0.0 \, \text {hr}$ to $1.0 \, \text {hr}$	$0.005 \, \text {hr}$

**Predictive performance.** For the CSTR case, representative values are $L=20$, $r=6$, with $\sigma$ determined by the same median-distance initialization followed by validation refinement. Regularization parameters $(\lambda _1,\lambda _2)$ are selected using the validation protocol described at the beginning of this section.

**Fig. 5:**
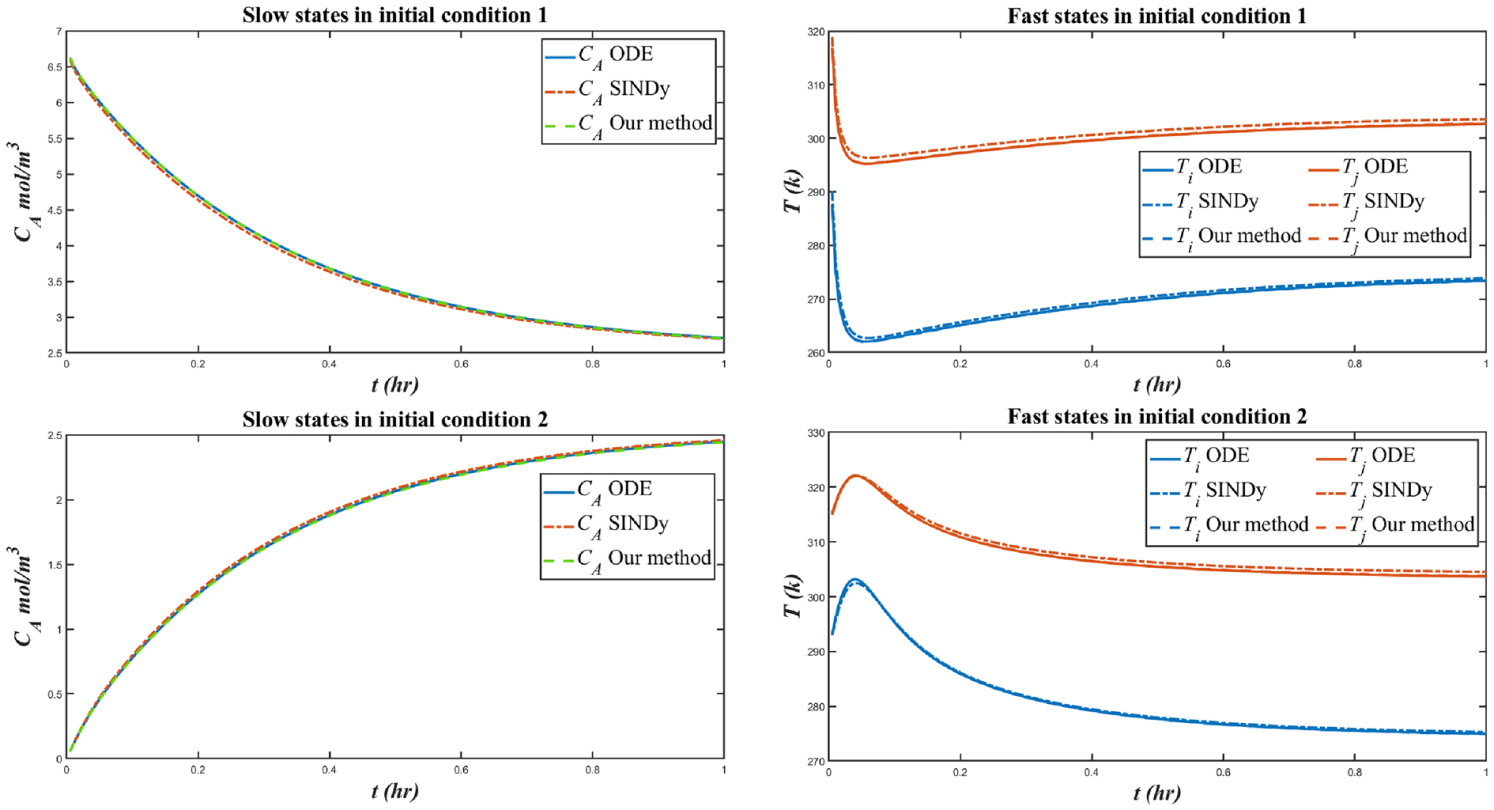
Long-horizon rollout prediction under clean (noise-free) simulated data for the Non-isothermal CSTR. Ground truth trajectories are compared with predictions obtained using the proposed method and SINDy.

Figure 5 and Table 7 summarize the prediction accuracy of the evaluated methods. For the slowly varying concentration $C_A$, all approaches achieve comparable performance under noise-free conditions. However, larger discrepancies arise in the temperature variables, particularly during the initial fast transient phase. Standard SINDy applied directly to raw measurements tends to accumulate error when temperature dynamics change rapidly, especially under $10\%$ noise.

The proposed pipeline maintains more stable tracking of both $T_r$ and $T_j$ across noise levels. This improvement is attributed to the combination of adaptive resampling, delay embedding, and latent-space regression, which collectively reduce sensitivity to short-term fluctuations while preserving dominant dynamical patterns. Notably, the performance gap widens as noise increases, indicating enhanced robustness under measurement perturbations.

**Fig. 6:**
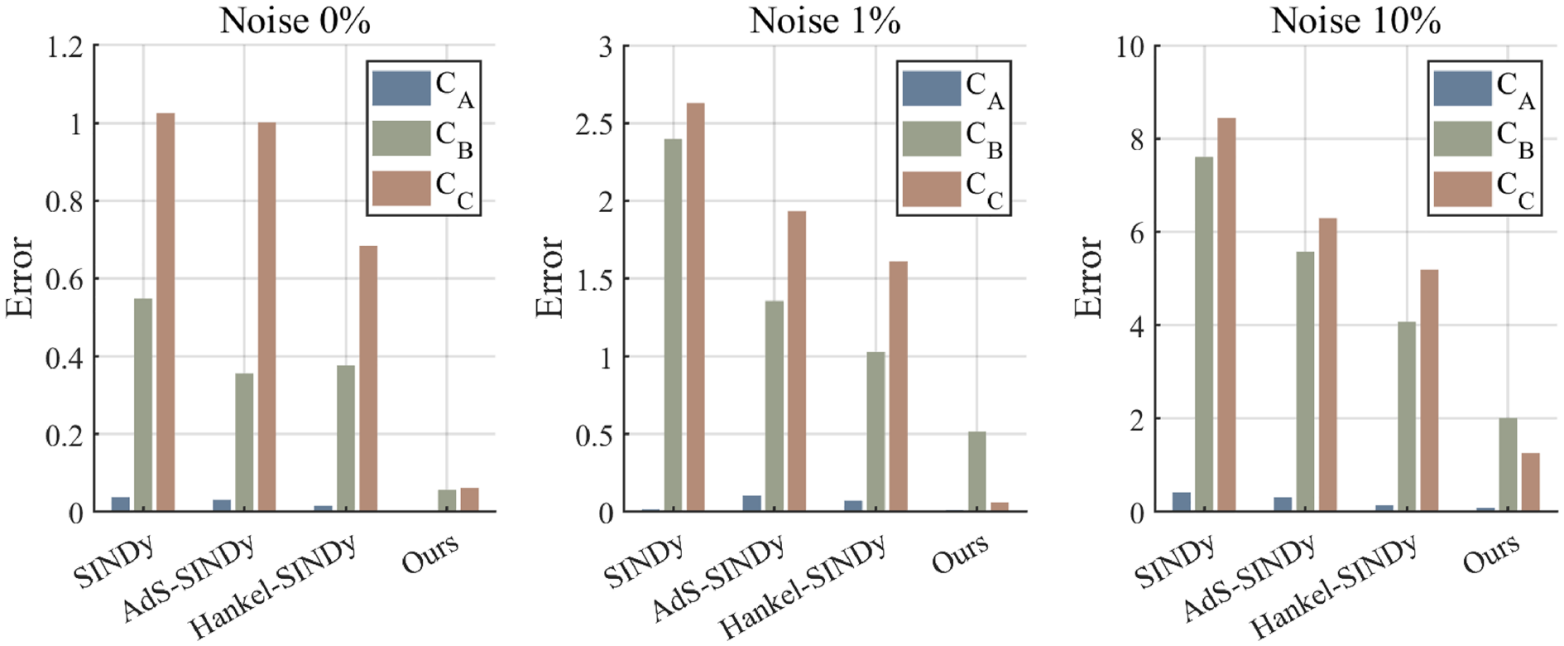
Error comparison of different identification methods under different noise levels for the Non-isothermal CSTR.

Overall, this nonlinear, thermally coupled example indicates the applicability of the proposed reduced-order modeling framework to systems exhibiting both strong state coupling and heterogeneous temporal scales. Detailed component-wise ablation and robustness analyses are provided in Sections “Ablation study” and "Generalization and robustness analysis" (Fig. 6).

**Table 7:** Error comparison for different methods under different initial conditions.

Method	Error (noise 0%)			Error (noise 1%)			Error (noise 10%)		
	$C_A$	$T_r$	$T_j$	$C_A$	$T_r$	$T_j$	$C_A$	$T_r$	$T_j$
Initial Condition 1									
SINDy	0.038	0.548	1.025	0.018	2.398	2.629	0.415	7.604	8.441
AdS-SINDy	0.031	0.356	1.001	0.107	1.356	1.931	0.311	5.573	6.289
Hankel-SINDy	0.016	0.377	0.684	0.072	1.028	1.608	0.142	4.067	5.184
**Ours**	**0.004**	**0.057**	**0.062**	**0.013**	**0.517**	**0.062**	**0.083**	**2.012**	**1.263**
Initial Condition 2									
SINDy	0.045	0.603	1.185	0.023	2.567	2.802	0.437	7.892	8.763
AdS-SINDy	0.035	0.410	1.125	0.112	1.432	2.023	0.329	5.742	6.478
Hankel-SINDy	0.019	0.412	0.732	0.082	1.116	1.685	0.154	4.231	5.345
**Ours**	**0.006**	**0.065**	**0.072**	**0.016**	**0.542**	**0.072**	**0.096**	**2.123**	**1.341**

### Grinding and classification process

**Fig. 7:**
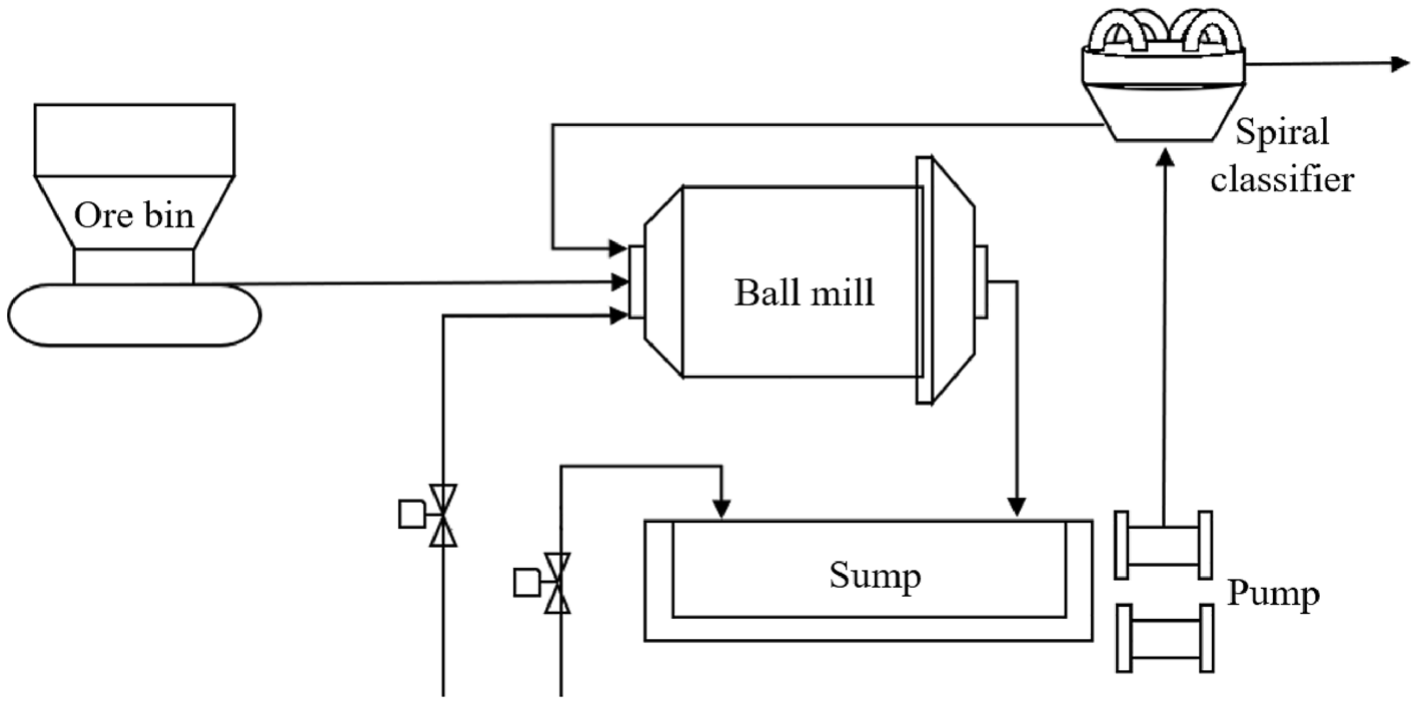
The grinding and classification process of the wet ball mill.

The final case study considers a real-world grinding and classification circuit in a wet ball milling process, as illustrated in Fig. 7^27^. The dataset was collected from an industrial beneficiation plant between December 15, 2021 and February 1, 2022, with a fixed sampling interval of 30 seconds. The recorded time series consists of 12 operational variables, including ore feed rate, water addition flow rate, hydrocyclone feed pressure, sump level, mill current, and related process measurements. To ensure comparability across variables with significantly different physical scales, all industrial signals are standardized prior to model identification. Specifically, each variable is transformed using its mean and standard deviation computed on the training segment only. This normalization prevents dominance of large-magnitude variables and avoids information leakage from the test data.

**Data description and prediction task.** Among the recorded variables, two representative output variables are selected for quantitative evaluation: the overflow concentration $C_{1o}$ and the overflow mass flow $M_{2o}$, which are key indicators of classification efficiency and downstream separation performance. All 12 variables are used as inputs for model identification.

The dataset is chronologically divided into a training segment (first 70% of the time series) and a testing segment (remaining 30%), ensuring that model evaluation is performed on future data not used during identification. Multi-step rollout prediction is conducted over the test segment. Since the industrial measurements inherently contain sensor noise and operational fluctuations, no artificial noise is injected in this example.

**Multiscale characteristics.** The grinding-classification circuit exhibits heterogeneous temporal behavior. Short-term fluctuations arise from feed disturbances and pump pressure variations on the order of minutes, whereas slower variations in sump level and concentration reflect longer material residence and accumulation effects. These interacting time scales coexist within the same trajectory, posing challenges for fixed-rate regression directly on raw signals.

**Predictive performance.** For the industrial dataset, representative values are $L=25$, $r=8$, with $\sigma$ determined using the same validation-based strategy. Regularization parameters are selected within the training subset following the unified protocol described above.

**Fig. 8:**
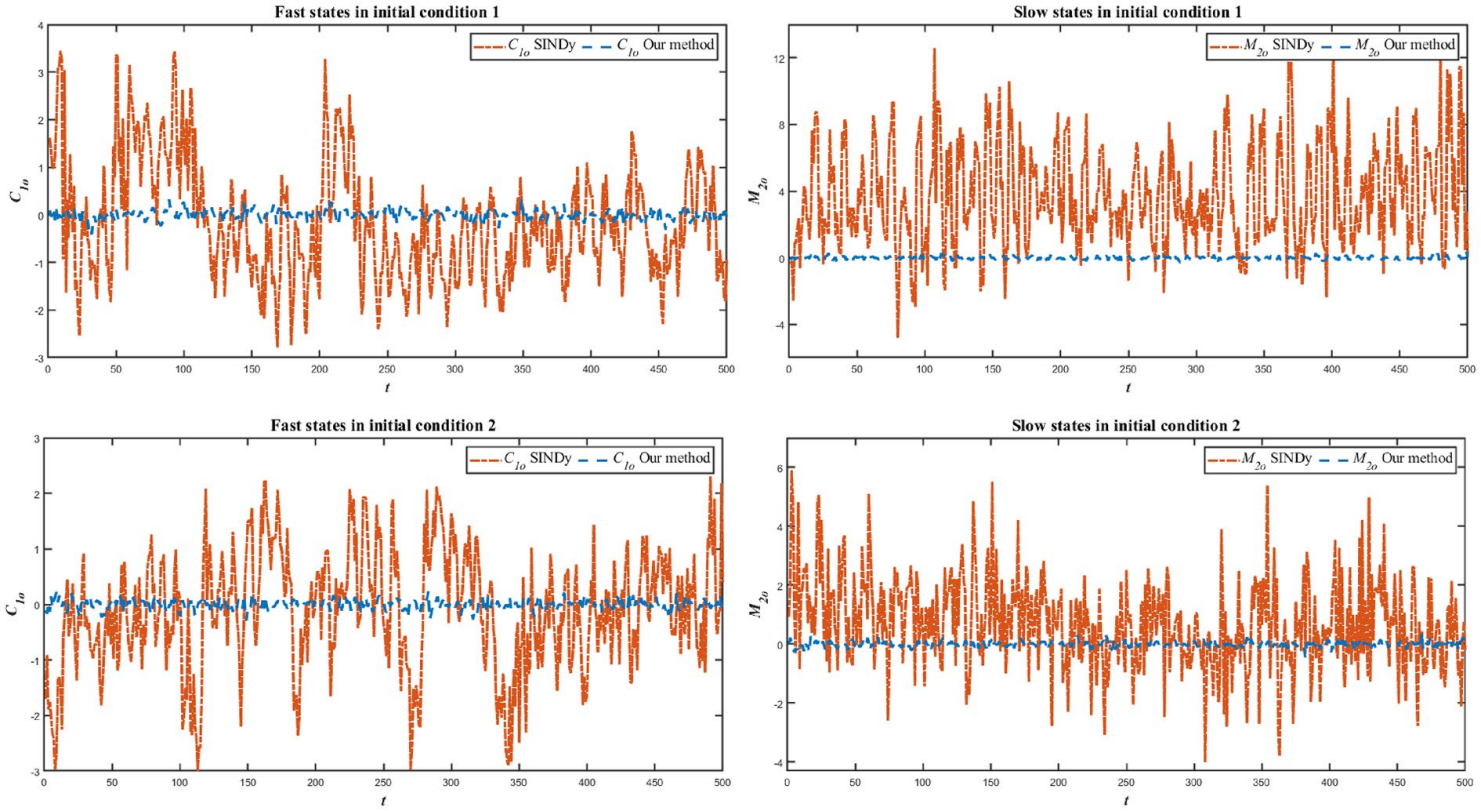
Mean squared estimation error (MSE) comparison between the proposed method and SINDy for the real grinding–classification industrial system (Example 3). The figure reports quantitative error metrics rather than raw process trajectories.

Figure 8 compares representative prediction trajectories obtained by the proposed method and baseline approaches, and quantitative results are summarized in Table 8. Standard SINDy applied to the raw measurements exhibits noticeable deviation during periods of rapid fluctuation, particularly in $M_{2o}$. Incorporating either adaptive sampling or Hankel embedding partially improves stability.

The proposed pipeline achieves lower mean-square prediction errors for both $C_{1o}$ and $M_{2o}$ on the test segment. The improvement is more pronounced for the more rapidly varying variable $M_{2o}$, indicating enhanced robustness in capturing short-term dynamics while maintaining stability over longer horizons.

Unlike the simulated benchmarks, this example involves unmodeled disturbances, process nonlinearities, and measurement imperfections inherent to industrial operation. The results demonstrate that the proposed reduced-order modeling framework remains numerically stable and predictive under real data conditions. Detailed ablation analysis, robustness evaluation under varying data lengths, and computational cost comparisons are presented in Sections “Ablation study”–"Computational efficiency analysis".

**Table 8:** Normalized mean-square error (NMSE) on the test segment for the industrial grinding dataset.

Method	Error		Method	Error	
	$C_{1o}$	$M_{2o}$		$C_{1o}$	$M_{2o}$
SINDy	0.184	3.711	Hankel-SINDy	0.108	1.413
AdS-SINDy	0.127	2.696	**Ours**	**0.012**	**0.021**

### Ablation study

To evaluate the contribution of individual components in the proposed pipeline, we conduct an ablation study on the non-isothermal CSTR system under $1\%$ noise, using the same training/testing protocol as in Section "Non-isothermal CSTR with jacket". Only the pipeline structure is modified, while all hyperparameters and data splits remain unchanged.

#### Ablation variants

The following reduced variants are considered:

- **Full model**: complete pipeline including adaptive resampling, spline smoothing, block Hankel embedding, kernel-based dimensionality reduction, and sparse regression in the latent space.
**–Resampling**: adaptive resampling is removed; identification is performed on uniformly sampled data, while keeping spline smoothing, Hankel embedding, kernel reduction, and latent regression.
**–Spline**: spline-based smoothing is replaced by finite-difference differentiation, while retaining other components.
**–Hankel**: delay embedding is removed; regression is performed directly on instantaneous states (with kernel reduction disabled accordingly).
**–Kernel**: block Hankel embedding is retained, but dimensionality reduction is performed using linear SVD instead of Gaussian kernel mapping.

#### Quantitative comparison

Table 9 reports the mean absolute prediction error (MAE) on the test segment for the CSTR system under $1\%$ noise (Initial Condition 1 shown for brevity; similar trends are observed for other initial conditions).

**Table 9:** Ablation study on the non-isothermal CSTR system (noise $1\%$, test segment MAE).

Variant	$C_A$	$T_r$	$T_j$
Full model	**0.013**	**0.517**	**0.062**
–Resampling	0.018	0.694	0.101
–Spline	0.021	0.812	0.134
–Hankel	0.024	1.146	0.228
–Kernel	0.017	0.631	0.094

#### Component-wise interpretation

Removing any individual component results in a consistent degradation of prediction accuracy. Eliminating adaptive resampling primarily affects the reconstruction of fast thermal transients, leading to increased temperature errors. Replacing spline smoothing with finite-difference differentiation increases sensitivity to noise and slightly amplifies error accumulation. Removing delay embedding produces the most significant deterioration, indicating that temporal aggregation plays a central role in stabilizing regression. Replacing kernel reduction with linear SVD leads to moderate performance loss, suggesting that nonlinear feature extraction contributes to robustness but is not solely responsible for the overall improvement.

#### Integration effect

Importantly, no single component alone reproduces the performance of the full model. While individual modules provide partial gains, the combination of adaptive resampling, temporal embedding, nonlinear dimensionality reduction, and latent sparse regression yields the lowest overall error. These results support the claim that the proposed framework is not merely a composition of independent mature techniques, but a coordinated integration in which each component addresses a specific numerical or structural challenge in multiscale time-series modeling.

### Generalization and robustness analysis

To further assess the robustness of the proposed framework beyond the nominal evaluation setting, we conduct additional experiments on the non-isothermal CSTR system under $1\%$ noise. All results are reported on unseen test data using multi-step rollout prediction.

#### Prediction horizon sensitivity

We first examine the influence of prediction horizon by extending the rollout length to $1\times$, $2\times$, and $4\times$ the original test horizon. Table 10 reports the corresponding mean absolute errors (Initial Condition 1).

As expected, error accumulates with increasing rollout length. However, the growth remains gradual and stable, without abrupt divergence, indicating that the identified latent dynamics preserve reasonable long-term behavior.

#### Training data reduction

To evaluate sensitivity to data availability, we vary the fraction of training data to $100\%$, $50\%$, and $25\%$ of the original training segment. Results are summarized in Table 10.

**Table 10:** Influence of prediction horizon and training data size on CSTR test error (noise $1\%$).

**Prediction horizon**				**Training data size**			
Setting	$C_A$	$T_r$	$T_j$	Setting	$C_A$	$T_r$	$T_j$
$1\times$	0.013	0.517	0.062	100%	0.013	0.517	0.062
$2\times$	0.021	0.694	0.108	50%	0.019	0.643	0.089
$4\times$	0.036	0.982	0.214	25%	0.028	0.811	0.137

Reducing the training data leads to moderate degradation in accuracy, yet the model remains stable and predictive even with limited data, suggesting that the latent representation captures dominant dynamics efficiently.

#### Initial-condition generalization

Finally, we assess generalization across initial conditions by training the model on a subset of initial conditions and evaluating it on previously unseen conditions within the admissible operating range. The resulting test errors remain within the same order of magnitude as those reported in Table 7, indicating that the learned latent dynamics are not restricted to a single trajectory but generalize across moderate variations in initial states.

Overall, these additional experiments indicate that the proposed framework exhibits stable multi-step rollout behavior, robustness to reduced training data, and reasonable generalization across operating conditions. Limitations under more extreme regime shifts are discussed in Section "Discussion and limitations".

### Computational efficiency analysis

Although the primary objective of the proposed framework is predictive accuracy under multiscale dynamics, computational efficiency is also relevant for reduced-order surrogate modeling. We therefore report representative training and inference costs for the non-isothermal CSTR system under $1\%$ noise. All experiments were conducted on the workstation described in the Methods section.

**Table 11:** Training and inference time comparison for the CSTR system (noise $1\%$).

Method	Training time (s)	Inference time (s)
SINDy	0.42	0.19
Hankel-SINDy	0.78	0.27
Proposed method	1.63	0.24

The proposed pipeline introduces moderate additional offline training cost due to adaptive resampling and kernel-based dimensionality reduction. However, once the latent dynamical system is identified, the multi-step rollout cost remains comparable to that of baseline methods. The identified latent model contains 6 nonzero terms (out of 45 candidate functions), yielding a compact reduced-order representation suitable for efficient prediction.

### Model interpretability and identified dynamics

A central motivation of sparse regression–based modeling is the recovery of compact dynamical representations that remain interpretable at the level of functional structure. Although the proposed framework performs regression in a latent coordinate space, the resulting model remains an explicit sparse dynamical system of the form

29 $$\begin{aligned} \dot{\boldsymbol{z}} = \Theta (\boldsymbol{z}) \boldsymbol{\Xi }, \end{aligned}$$

where $\Theta (\boldsymbol{z})$ is a predefined library and $\boldsymbol{\Xi }$ is a sparse coefficient matrix.

#### Sparsity structure

For the non-isothermal CSTR system (noise $1\%$), the identified latent model contains 6 nonzero terms out of 45 candidate functions. Table 12 lists the dominant terms for one representative initial condition.

**Table 12:** Dominant nonzero terms in the identified latent CSTR model (noise $1\%$).

Term	Coefficient magnitude	Relative rank
$z_1$	0.84	1
$z_2$	0.63	2
$z_1 z_2$	0.41	3
$z_2^2$	0.27	4
$z_3$	0.19	5
$z_1 z_3$	0.12	6

The limited number of active terms indicates that the latent dynamics admit a compact representation, despite the nonlinear coupling present in the original reactor equations.

#### Stability across initial conditions

To evaluate structural consistency, we compare the support (set of nonzero terms) identified across different initial conditions. The overlap ratio of active terms exceeds $80\%$ between independently trained models, indicating that the dominant functional structure is not restricted to a single trajectory.

#### Comparison with direct SINDy

For comparison, direct SINDy applied to the original state variables under the same noise level produces a larger number of active terms (typically 12–15 out of 45 candidates), with coefficients exhibiting higher variance across initial conditions. This increased density reflects sensitivity to noise and multiscale transient effects.

In contrast, regression in the delay-embedded latent space yields a more parsimonious model while maintaining predictive performance. Although the latent coordinates do not correspond directly to physical state variables, the resulting model remains an explicit sparse dynamical system that can be analyzed, simulated, and inspected term by term.

Overall, the proposed framework preserves the structural interpretability associated with sparse regression, while improving numerical stability in multiscale regimes.

### Discussion and limitations

The proposed framework is designed for predictive reduced-order modeling of nonlinear systems under fixed-rate sampling and moderate noise conditions, with an emphasis on numerical stability and compact representation. The empirical results indicate improved robustness in the presence of heterogeneous temporal responses. Nevertheless, the applicability of the approach is subject to several limitations that define its current scope.

First, the notion of multiscale behavior considered in this work is primarily temporal. The formulation does not explicitly account for systems with strongly coupled spatial–temporal dynamics or extreme stiffness across multiple physical processes. In such cases, where spatial interactions or multi-physics coupling dominate system evolution, the present representation may be insufficient. Extending the framework to these settings would likely require additional structural components, such as operator-based formulations or spatially informed representations.

Second, the method relies on a predefined candidate function library in the latent space. Although sparse regression enforces parsimony, the expressiveness and conditioning of the resulting model depend on the quality of this library. An insufficient set of candidate functions may limit representational capacity, while an overly rich library can introduce collinearity and increase sensitivity to regularization. As a result, performance may vary across systems with different underlying dynamics, and systematic or adaptive library construction remains an important direction for further improvement.

Third, the current formulation is developed in an offline setting. Key preprocessing steps, including adaptive resampling, spline-based smoothing, and kernel-based reduction, are performed prior to model identification. This limits direct applicability to streaming or real-time environments, where data arrive sequentially and system conditions may evolve. Extending the framework to online scenarios would require incremental updating strategies or recursive identification mechanisms.

Fourth, the latent-space formulation improves numerical conditioning but does not preserve a direct correspondence with physical state variables. The identified model therefore remains interpretable at the level of sparse functional structure in the latent space, but does not guarantee recovery of first-principles governing equations. This reflects a deliberate modeling choice: the framework prioritizes predictive reduced-order representation over explicit mechanistic interpretability, which may limit its use in applications requiring direct physical insight.

Finally, robustness has been evaluated under variations in initial conditions and moderate noise levels. Performance under more challenging conditions, such as abrupt regime shifts, previously unseen operating regions, or severe measurement corruption, remains less certain. In such scenarios, particularly for systems with rapidly changing operating modes, additional mechanisms such as adaptive updating or regime-switching strategies may be required.

Within these boundaries, the results suggest that the integration of adaptive resampling, temporal embedding, nonlinear dimensionality reduction, and latent-space sparse regression provides a coherent strategy for stabilizing regression in multiscale industrial time-series settings, where direct modeling in the original space is often sensitive to noise and sampling imbalance.

## Conclusion

This work investigated predictive reduced-order modeling for nonlinear dynamical systems exhibiting heterogeneous temporal behavior under fixed-rate sampling. In such settings, direct sparse regression on raw measurements is often sensitive to fast transients, sampling imbalance, and measurement noise, which can degrade long-horizon predictive performance. To address these challenges, we developed a structured data-driven framework that combines adaptive resampling, spline-based smoothing, delay embedding, kernel-based dimensionality reduction, and sparse regression in a latent coordinate space. This formulation transforms the identification problem into a better-conditioned regression task, in which dominant temporal patterns are preserved while noise and redundancy are attenuated. Empirical evaluations on representative reactor systems and an industrial time-series dataset demonstrate that the proposed approach provides improved predictive robustness and numerical stability compared with direct sparse regression, particularly in the presence of multiscale temporal dynamics and moderate noise. Component-wise analysis further indicates that temporal embedding and structured preprocessing play a central role in stabilizing regression, while latent representations improve conditioning without substantially increasing model complexity. The resulting models are defined in a reduced latent space and are intended as predictive surrogates rather than explicit reconstructions of governing equations. This design reflects a deliberate trade-off between numerical stability and physical interpretability, and clarifies the scope of applicability of the proposed framework.

Overall, the results indicate that combining temporal embedding with latent-space sparse regression provides a structured and practical approach for predictive reduced-order modeling of industrial time-series systems under realistic data constraints. Future work will explore extensions to online updating, adaptive library construction, and broader classes of multiscale dynamical systems.

## Data Availability

Code and data will be available from the authors upon a reasonable request.

## Abbreviations

$\boldsymbol{x}(t)$: Original state vector at time *t*
$\mathscr {D}_{\textrm{adapt}}$: Adaptively resampled dataset
*L*: Delay embedding window length
*P*: Number of delay-embedded snapshots
$\tilde{\boldsymbol{K}}$: Centered kernel matrix
$\boldsymbol{z}(t)$: Latent state vector
$\boldsymbol{\Xi }$: Sparse coefficient matrix
$\mathscr {D}$: Original sampled dataset $\{\boldsymbol{x}(t_k)\}_{k=1}^N$
$$\Delta t$$: Sampling interval
$\hat{\mathscr {H}}$: Block Hankel matrix in $\mathbb {R}^{dL \times P}$
$\boldsymbol{K}$: Kernel Gram matrix
$\boldsymbol{Z}$: Latent coordinate matrix in $\mathbb {R}^{r \times P}$
$\Theta (\boldsymbol{z})$: Candidate function library in latent space
$\dot{\boldsymbol{z}}$: Time derivative of latent state
